# Measurement of Amniotic Fluid Interleukin-6 Using Commercial Kits

**DOI:** 10.1155/S1064744997000379

**Published:** 1997

**Authors:** Tsunenobu Tamura, William W. Andrews, Kelley E. Johnston, G. Philamon Hemstreet, Robert L. Goldenberg

**Affiliations:** 1 Department of Nutrition Sciences 218 Webb Building University of Alabama at Birmingham Birmingham AL USA; 2 Department of Obstetrics and Gynecology University of Alabama at Birmingham Birmingham AL USA

## Abstract

*Objective:* The association between increased amniotic fluid interleukin-6 (IL-6) concentrations and preterm labor has received increasing attention. Several research groups have evaluated this association using commercial IL-6 kits, which principally use the sandwich-enzyme-immunoassay method, and were originally created to measure IL-6 in plasma, serum, or culture media. We evaluated commercial kits for the determination of IL-6 in amniotic fluid.

*Methods:* Seven commercial kits were used to determine IL-6 concentrations in three amniotic fluid samples which were obtained from patients with clinical chorioamnionitis during labor and five from normal pregnancies at mid-trimester.

*Results:* Amniotic fluid IL-6 values differed significantly with some having over a 50-fold discrepancy and the recovery of known IL-6 added to amniotic fluid ranged from 12 to 123%. However, by all kits we were able to identify that amniotic fluid from patients with chorioamnionitis contained significantly higher IL-6 concentrations than those from normal mid-trimester pregnancies.

*Conclusion:* Our data indicate that standardization of the method for measuring IL-6 in amniotic fluid is desirable for the comparison of values from various laboratories.


Infectious Diseases in Obstetrics and Gynecology 5:222–225 (1997)
© 1997 Wiley-Liss, Inc.

### Measurement of Amniotic Fluid Interleukin-6 Using Commercial Kits

**Tsunenobu Tamura,[1]* William W. Andrews,[2] Kelley E. Johnston,[1] G. Philamon Hemstreet,[2] and Robert L. Goldenberg[2]**

[1]*Department of Nutrition Sciences, University of Alabama at Birmingham, Birmingham, AL*
[2]*Department of Obstetrics and Gynecology, University of Alabama at Birmingham, Birmingham, AL*

ABSTRACT

***Objective:*** **The association between increased amniotic fluid interleukin-6 (IL-6) concentrations and preterm labor has received increasing attention. Several research groups have evaluated this association using commercial IL-6 kits, which principally use the sandwich-enzyme-immunoassay method, and were originally created to measure IL-6 in plasma, serum, or culture media. We evaluated commercial kits for the determination of IL-6 in amniotic fluid.**

***Methods:*** **Seven commercial kits were used to determine IL-6 concentrations in three amniotic fluid samples which were obtained from patients with clinical chorioamnionitis during labor and five from normal pregnancies at mid-trimester.**

***Results:*** **Amniotic fluid IL-6 values differed significantly with some having over a 50-fold discrepancy and the recovery of known IL-6 added to amniotic fluid ranged from 12 to 123%. However, by all kits we were able to identify that amniotic fluid from patients with chorioamnionitis contained significantly higher IL-6 concentrations than those from normal mid-trimester pregnancies.**

***Conclusion:*** **Our data indicate that standardization of the method for measuring IL-6 in amniotic fluid is desirable for the comparison of values from various laboratories. Infect. Dis. Obstet. Gynecol. 5:222–225, 1997.** © 1997 Wiley-Liss, Inc.

KEY WORDS

amniotic fluid; assay kits; chorioamnionitis; interleukin-6

Interleukin-6 (IL-6) is a multifunctional protein and an inducer of the acute-phase response. It is produced by a variety of cells, and its production is stimulated by infections.[1,2] Increasing information has become available on the association between amniotic fluid IL-6 concentrations and pregnancy outcome.[3–14] Several groups of investigators have used commercial kits for the measurement of IL-6 in amniotic fluid.[3,7,8,10–12,14] However, these kits were originally intended to measure IL-6 in plasma, serum, and tissue-culture media, not in amniotic fluid. To evaluate various kits for the measurement of IL-6 in amniotic fluid, we used seven commercially available kits and compared the results of the concentrations of amniotic fluid IL-6.

### MATERIALS AND METHODS

Amniotic fluid samples were obtained during labor using a transcervical intrauterine pressure catheter from three women with clinical chorioamnionitis. Clinical chorioamnionitis was diagnosed when all

Contract grant sponsor: NIH; Contract grant number: HD32901; Contract grant sponsor: Agency for Health Care Policy Research; Contract grant number: DHHS 290-92-0055.

*Correspondence to: Dr. Tsunenobu Tamura, Department of Nutrition Sciences, 218 Webb Building, University of Alabama at Birmingham, Birmingham, AL 35294-3360.

Received 19 July 1996

Accepted 17 February 1997

Clinical Study of the following were present; 1) a fever over 38°C; 2) uterine tenderness; 3) fetal tachycardia; 4) absence of another clinical source of infection; and 5) clinical diagnosis of chorioamnionitis by managing physicians with initiation of antimicrobial therapy. Five amniotic fluids were collected during genetic amniocentesis at about 17 weeks of gestation. These amniotic fluid samples were originally collected for various clinical evaluations and would have been discarded, if not used in this investigation. Amniotic fluid samples were centrifuged (900*g* for 10 min), separated into aliquots, and stored at −70°C until analyses.

Human IL-6 assay kits were obtained from seven commercial sources (BioSource, Camarillo, CA; Cistron, Pine Brook, NJ; Endogen, Cambridge, MA; Genzyme, Cambridge, MA; Immunotech, Westbrook, ME; PerSeptive Diagnostics, Cambridge, MA; and R&D Systems, Minneapolis, MN), all of which principally use the quantitative sandwich-enzyme-immunoassay method using 96-well microplates. These kits have slight differences not only in the ranges of standard provided but also the assay procedures and sample volumes (Table 1). For the measurement of optical density, a microplate reader (BioRad, Richmond, CA) was used at the appropriate wavelengths specified by the manufacturers.

The assay procedures were carried out according to the instructions by the manufacturers, and all assays were performed in duplicate. The dilution of the samples was made based on the instructions provided, and a pooled amniotic fluid was used as a diluent after the fluid was heated at 100°C for 10 min for the kits prepared by Cistron, Endogen, and PerSeptive Diagnostics as suggested by these manufacturers. The range of dilutions used in amniotic fluid samples was between 2- and 200-fold. Polypropylene tubes were used for the dilutions because it was pointed out in the instructions by two manufacturers (Immunotech and Cistron) that polystyrene and glass may adsorb IL-6. The calculation of the final concentrations was made using linear regression after logarithmic transformation of optical densities. Although some instructions indicated that we should use log-linear regression, the correlation coefficients using linear regression with a log-log scale were higher than those obtained by log-linear regression in all kits.

Evaluation of the recovery of a known amount of IL-6 added to an amniotic fluid sample was performed by adding an aliquot of the standard solution containing the highest concentration of IL-6 supplied by each manufacturer. The volumes of the standard solution used for this recovery test ranged from 25 to 50% of the amount of amniotic fluid samples in each well (300 μl). This recovery study was done once in duplicate using three levels of the standard. The agreement between duplicates for entire assays was evaluated by calculating the correlation coefficients between the optical density for each duplicate after logarithmic transformation. The evaluation of day-to-day variations was not performed for each kit in the present study.

### RESULTS

The source, standard range, and characteristics of the commercial kits are summarized in Table 1. Some kits were more labor intensive than others. As shown in Table 2, the IL-6 concentrations in amniotic fluid measured in the present study varied greatly depending on the kit used for the assay. The agreement between the duplicates was generally good (Pearson correlation coefficient of over 0.980) except for one kit (BioSource) for which the correlation coefficient was 0.923. The correlation coefficients of standard curves generated on a log-log scale were excellent with values over 0.950 for five kits, whereas two kits had correlation coefficients of only 0.738 and 0.882 (Immunotech and BioSource, respectively). The concentrations of IL-6 in amniotic fluid samples (sample 3) differed significantly with over a 50-fold discrepancy among the seven kits. Furthermore, the recovery of known IL-6 added to the amniotic fluid samples varied from 12 to 123% depending on the kits used (Table 2).

Despite these wide variations in IL-6 concentrations among the kits tested, all kits clearly distinguished higher amniotic fluid concentrations of IL-6 obtained from women with clinical chorioamnionitis during labor compared to those from women with normal pregnancies at mid-trimester. The mean of three samples from patients with chorioamnionitis measured by all seven kits was 20,500 ng/l with a range between 2,300 and 116,000 ng/l, while the mean of five samples from normal pregnancies was 461 ng/l with a range between 30 and 3,100 ng/l. IL-6 in amniotic fluid was unstable,

TABLE 1. Comparisons between seven commercially available IL-6 assay kits

| Kit | Standard range (ng/l) | Incubations | Diluent recommended | Wavelength (nm) | Sample volume (μl/well) | Calculation recommended |
|---|---|---|---|---|---|---|
| BioSource | 15.6–500 | 2 h + 1 h + 20 min | Provided | 450 | 50 | Not specified |
| Cistron | 12.5–1,000 | 20 min × 4 | AF[a] (boiled) | 450 | 100 | Log-log |
| Endogen | 39–1,050 | 1 h + 30 min × 2 | AF (boiled) | 450 minus 550 | 50 | Log-linear |
| Genzyme | 35–1,800 | 30 min × 2 + 15 min + 10 min | Provided | 450 | 50 | Log-linear |
| Immunotech | 3.9–1,000 | 2 h + 15 min | Provided | 404–414 | 100 | Log-linear |
| PerSeptive | 50–2,000 | 1 h + 30 min × 2 + 15 min | AF (boiled) | 450 | 100 | Log-linear |
| R & D | 3.13–300 | 2 h × 2 + 20 min | Provided | 450 minus 540 | 100 | Log-log |

[a]Amniotic fluid.

TABLE 2. IL-6 concentrations (ng/l) in amniotic fluid samples and assay performance in seven kits[a]

| Samples | BioSource | Cistron | Endogen | Genzyme | Immunotech | PerSeptive | R & D |
|---|---|---|---|---|---|---|---|
| 1 | 8,900 | 12,900 | 7,300 | 3,900 | 12,400 | 2,300 | 4,700 |
| 2 | 11,400 | 21,200 | 25,800 | 5,900 | 86,200 | 4,700 | 26,000 |
| 3 | 9,700 | 116,000 | 14,200 | 4,700 | 43,000 | 2,300 | 7,450 |
| 4 | 60 | 710 | 56 | 71 | 130 | 440 | 90 |
| 5 | 1,800 | 3,100 | 1,100 | 750 | 1,000 | 440 | 1,000 |
| 6 | ND[b] | 440 | ND | 90 | 80 | ND | 30 |
| 7 | 210 | ND | 290 | 80 | ND | 40 | 140 |
| 8 | 60 | ND | 480 | 70 | ND | 30 | 130 |
| Recovery (%) | 69 | 77 | 85 | 123 | 111 | 12 | 95 |
| Duplicate agreement[c] | 0.923 | 0.985 | 0.996 | 0.999 | 0.999 | 0.996 | 0.993 |
| Linear regression[d] | 0.545 | 0.947 | 0.966 | 0.952 | 0.778 | 0.970 | 0.934 |

[a]Data are calculated based on the mean of duplicates.
[b]Not determined due to insufficient amount of samples due to repeated freeze-thaw.
[c]Correlation coefficients between duplicate optical density readings.
[d]Correlation coefficients of linear regression for standard curves after both optical density and standard concentrations were log-transformed.

since the concentrations of IL-6 decreased markedly once the samples had been thawed and refrozen at –70°C. Therefore, several dilutions of amniotic fluid should be included at the initial assay to avoid additional freeze-thaw cycles which apparently destroy labile IL-6. In addition, the assay procedures for each kit took a maximum of 7 h including calculations. Therefore, the data can be reported within 24 h to physicians who manage patients.

### DISCUSSION

Because of strong interest in establishing the association between genital tract infections and pregnancy outcome,[3–14] many investigators, using commercially available kits, have sought to measure IL-6 concentrations in amniotic fluid samples.[3,7,8,10–12,14] However, it has not been well established whether the commercially available kits are suitable to measure IL-6 in amniotic fluid samples, because these kits were originally created for the measurement of IL-6 in plasma, serum, or tissue-culture media. Our data indicate that commercially available kits can be successfully used to measure IL-6 in amniotic fluid, since it was possible to distinguish the samples with high IL-6 levels from those with low IL-6 levels with all kits used in this study.

However, the values obtained using the seven kits in this study were quite different, indicating that it is unreasonable to compare values obtained using different kits in various laboratories. There are several possible explanations for these discrepancies among the kits. These include: 1) differences in the antibodies to IL-6 among kits which may have different responses to allotropes of IL-6 produced by various tissues, as pointed out by Bienvenu et al.[15]; 2) the protein matrix in amniotic fluid which affects antibody reactivity to IL-6 in assay kits originally prepared for the measurement of IL-6 in plasma, serum, or culture media; and 3) differences in the preparation of the standard used in each kit. No instruction stated how the concentrations of the standard were calibrated for the preparation of the kits.

In conclusion, it is essential that normal ranges be established for IL-6 concentrations in amniotic fluid for each kit used in each laboratory. In order to compare the values from various laboratories, it is desirable to standardize the method to measure IL-6 in amniotic fluid.

### REFERENCES

1. Van Snick J: Interleukin-6: An overview. Annu Rev Immunol 8:253–278, 1990.
2. Heinrich PC, Castell JV, Andus T: Interleukin-6 and the acute phase response. Biochem J 265:621–636, 1990.
3. Hiller SL, Witkin SS, Krohnn MA, Watts DH, Kiviat NB, Eschenbach DA: The relationship of amniotic fluid cytokines and preterm delivery, amniotic fluid infection, histologic chorioamnionitis, and chorioamnion infection. Obstet Gynecol 81:941–948, 1993.
4. Romero R, Yoon BH, Kenney JS, Gomez R, Allison AC, Sehgal PB: Amniotic fluid interleukin-6 determinations are of diagnostic and prognostic value in preterm labor. Am J Reprod Immunol 30:167–183, 1993.
5. Romero R, Yoon BH, Mazor M, et al.: A comparative study of the diagnostic performance of amniotic fluid glucose, white blood cell count, interleukin-6, and Gram stain in the detection of microbial invasion in patients with preterm premature rupture of membranes. Am J Obstet Gynecol 169:839–851, 1993.
6. Romero R, Yoon BH, Mazor M, et al.: The diagnostic and prognostic value of amniotic fluid white blood cell count, glucose, interleukin-6, and Gram stain in patients with preterm labor and intact membranes. Am J Obstet Gynecol 169:805–816, 1993.
7. Greig PC, Ernest JM, Teot L, Erikson M, Talley R: Amniotic fluid interleukin-6 levels correlate with histologic chorioamnionitis and amniotic fluid cultures in patients in premature labor with intact membranes. Am J Obstet Gynecol 169:1035–1044, 1993.
8. Cox SM, Roberts S, Roussis P, Campbell BA, Curry TE Jr: Prematurity, subclinical intraamniotic infection, and fetal biophysical parameters: Is there a correlation? Infect Dis Obstet Gynecol 1:76–81, 1993.
9. Saito S, Kasahara T, Kato Y, Ishihara Y, Ichijo M: Elevation of amniotic fluid interleukin 6 (IL-6), IL-8 and granulocyte colony stimulating factor (G-CSF) in term and preterm parturition. Cytokine 5:81–88, 1993.
10. Silver RM, Schwinzer B, McGregor JA: Interleukin-6 levels in amniotic fluid in normal and abnormal pregnancies: Preeclampsia, small-for-gestational-age fetus, and premature labor. Am J Obstet Gynecol 169:1101–1105, 1993.
11. Dudley DJ, Hunter C, Mitchell MD, Varner MW: Clinical value of amniotic fluid interleukin-6 determinations in the management of preterm labour. Br J Obstet Gynaecol 101:592–597, 1994.
12. Coultrip LL, Lien JM, Gomez R, Kapernick P, Khoury A, Grossman JH: The value of amniotic fluid interleukin-6 determination in patients with preterm labor and intact membranes in the detection of microbial invasion of the amniotic cavity. Am J Obstet Gynecol 171:901–911, 1994.
13. Andrews WW, Hauth JC, Goldenberg RL, Gomez R, Romero R, Cassell GH: Amniotic fluid interleukin-6: Correlation with upper genital tract microbial colonization and gestational age in women delivered after spontaneous labor versus indicated delivery. Am J Obstet Gynecol 173:606–612, 1995.
14. Wenstrom KD, Andrews WW, Tamura T, DuBard MB, Johnston KE, Hemstreet GP: Elevated amniotic fluid interleukin-6 levels at genetic amniocentesis predict subsequent pregnancy loss. Am J Obstet Gynecol 175:830–833, 1996.
15. Bienvenu J, Coulon L, Doche C, Gutowski M-C, Grau GE: Analytical performances of commercial ELISA-kits for IL-2, IL-6 and TNF-α. A WHO study. Eur Cytokine Netw 4:447–451, 1993.
